# ITRAQ-based proteomic analysis reveals possible target-related proteins in human adrenocortical adenomas

**DOI:** 10.1186/s12864-019-6030-5

**Published:** 2019-08-16

**Authors:** He Ma, Ranwei Li, Xin Di, Xin Jin, Yan Wang, Bingjie Lai, Cailian Shi, Mingxin Ji, Xinran Zhu, Ke Wang

**Affiliations:** 1 Department of Respiratory Medicine, the Second Hospital of Jilin University, Changchun, China; 2 Department of Urinary Surgery, the Second Hospital of Jilin University, Changchun, China; 3 Department of Hematology, the Second Hospital of Jilin University, Changchun, China; 4 Department of Intensive Care Unit, the Second Hospital of Jilin University, Changchun, China; 5 Department of Anesthesiology, the Second Hospital of Jilin University, Changchun, China

**Keywords:** iTRAQ, Adrenocortical adenoma, Proteomics, Differentially expressed protein

## Abstract

**Background:**

Adrenocortical adenomas (ACAs) can lead to the autonomous secretion of aldosterone responsible for primary aldosteronism (PA), which is the most common form of secondary arterial hypertension. However, the authentic fundamental mechanisms underlying ACAs remain unclear.

**Objective:**

Isobaric tags for relative and absolute quantitation (iTRAQ)-based proteomics and bioinformatics analyses from etiological studies of ACAs were performed to screen the differentially expressed proteins (DEPs) and investigate the relevant mechanisms of their occurrence and development. Results could help determine therapeutic targets of clinical significance.

**Methods:**

In the present study, iTRAQ-based proteomics was applied to analyze ACA tissue samples from normal adrenal cortex tissues adjacent to the tumor. Using proteins extracted from a panel of four pairs of ACA samples, we identified some upregulated proteins and other downregulated proteins in all four pairs of ACA samples compared with adjacent normal tissue. Subsequently, we predicted protein–protein interaction networks of three DEPs to determine the authentic functional factors in ACA.

**Results:**

A total of 753 DEPs were identified, including 347 upregulated and 406 downregulated proteins. The expression of three upregulated proteins (E2F3, KRT6A, and ALDH1A2) was validated by Western blot in 24 ACA samples. Our data suggested that some DEPs might be important hallmarks during the development of ACA.

**Conclusions:**

This study is the first proteomic research to investigate alterations in protein levels and affected pathways in ACA using the iTRAQ technique. Thus, this study not only provides a comprehensive dataset on overall protein changes but also sheds light on its potential molecular mechanism in human ACAs.

**Electronic supplementary material:**

The online version of this article (10.1186/s12864-019-6030-5) contains supplementary material, which is available to authorized users.

## Background

Primary aldosteronism (PA) is considered the most common cause of endocrine hypertension [1, 2]; it occurs in approximately 10–20% of hypertensive patients. Adrenocortical adenomas (ACAs) can lead to the autonomous secretion of aldosterone responsible for PA [3], which is the most frequent form of secondary arterial hypertension [4, 5]. Even though previous proteomic studies have already focused on differentially expressed proteins (DEPs) and made adequate progress in the understanding of the genetic bases of aldosterone- and cortisol-producing ACAs in the past few years [6–8], the authentic molecular mechanism and fundamental biological activities of DEPs underlying ACA remain ambiguous.

Additionally, quantitative proteomics, as an important methodology based on mass spectrometry, is widely used in the biological and clinical research of various diseases, such as the monitoring of specific disease biomarkers or the identification of functional modules and pathways [9–12]. Bioinformatic analysis of the dynamic transcriptome and expression regulation may guide future research on the mechanisms of ACA. Both isobaric tags for relative and absolute quantitation (iTRAQ) and label-free methods have been broadly applied for quantitative proteomics [13–16]. These techniques are compatible with high-throughput and high speed and can improve the reproducibility of prefractionation of complex peptide mixtures [17–19]. Nevertheless, proteomic studies about ACA are limited. Establishing differentially expressed protein–protein interaction (PPI) networks using bioinformatic data will lead to an improved understanding of the pathogenesis of ACA.

In this work, iTRAQ-based proteomic analysis was conducted based on the etiological study of adrenal adenoma to screen DEPs and explore the relevant mechanisms of its occurrence and development. Results of this study may be used to determine therapeutic targets of clinical significance, which might lay a theoretical foundation for the early diagnosis and effective treatment of adrenal adenoma.

## Results

In this study, iTRAQ was used to assess proteome changes between adrenocortical adenoma tissue and adjacent normal adrenal cortex tissue. On the basis of data acquisition, 753 DEPs were identified: 347 upregulated and 9406 downregulated proteins.

### Gene ontology (GO) analysis results

**Fig. 1:**
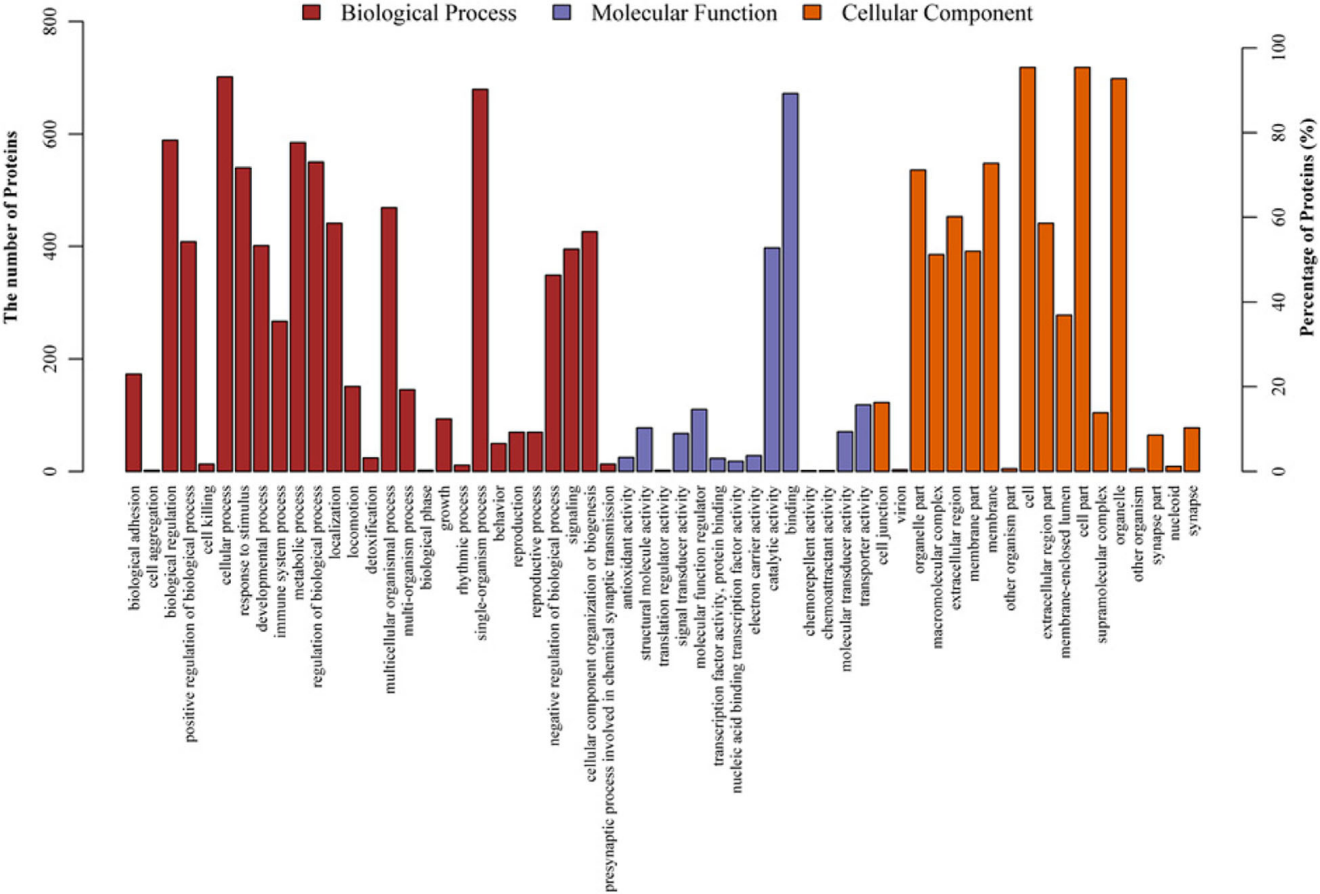
Gene Ontology (GO) analysis of differentially expressed proteins in adrenocortical adenomas compared with control. GO analysis was performed according three terms: Molecular Functions, Biological Process and Cellular Component

GO is a standardized functional classification system that provides a dynamically updated standardized vocabulary to describe the properties of genes and gene products in an organism from three perspectives: biological process, molecular function, and cell component [20] (Fig. 1).

**Fig. 2:**
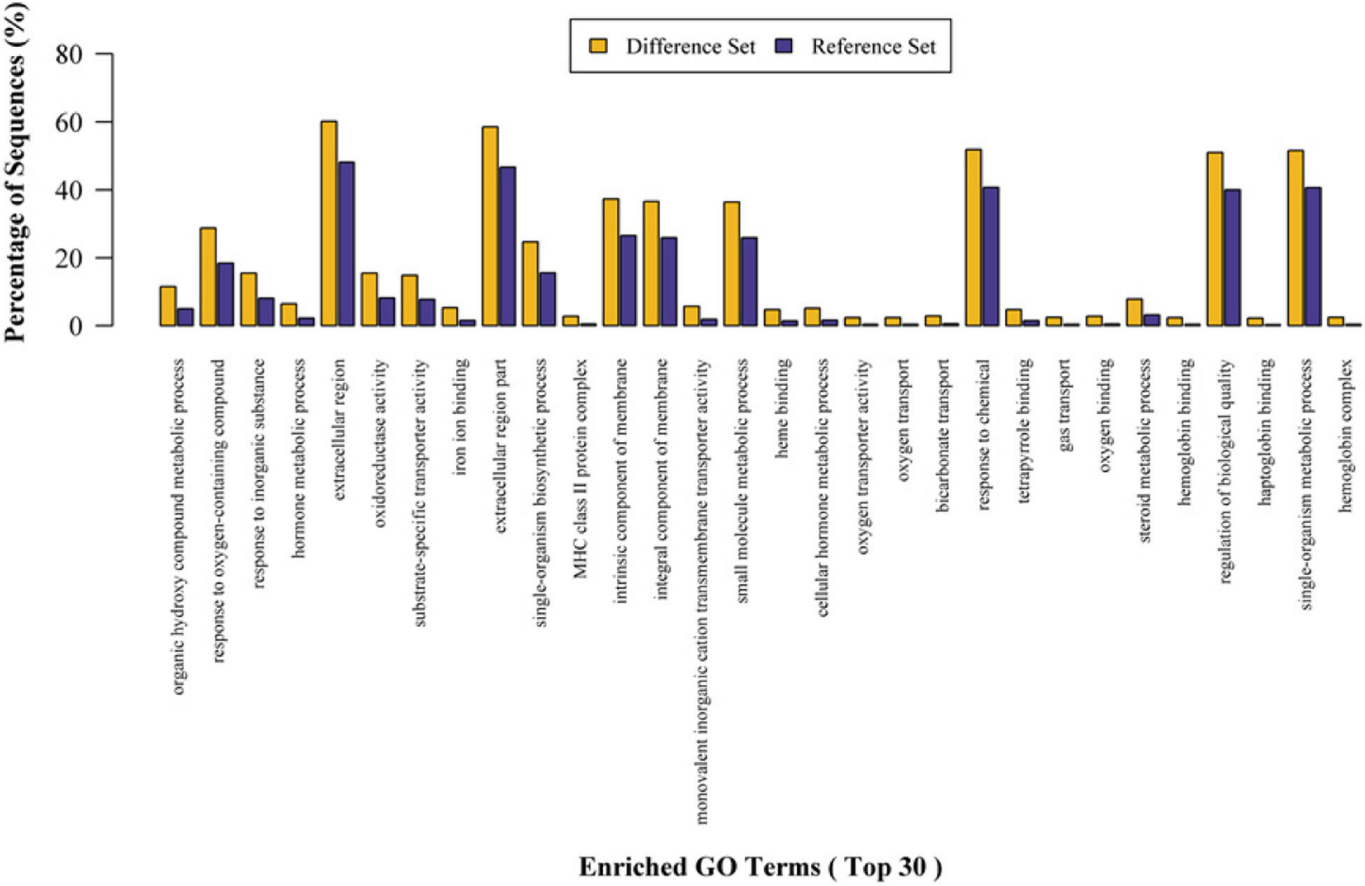
GO enrichment analysis (Difference set: target protein set; Reference set: background protein set)

The GO annotation of target proteins can classify these involved proteins in terms of biological process, molecular function, and cellular component (Fig. 2). Although the proportion of each classification can reflect the impact of biological factors on each classification in the experimental design to a certain extent, evaluations on the significance of each classification depending on the ratio alone are inaccurate. Notably, the distributions of each classification should be considered in overall protein collection, such as all qualitative proteins in an experiment or all known proteins of the species.

Among the 753 DEPs, 347 and 406 proteins were significantly upregulated and downregulated in ACA samples, respectively. The top 16 upregulated proteins included E2F3 protein (Table 1). Of the 16 proteins, keratin was the most upregulated protein, and its level was increased by 3.39-fold in ACA samples. Conversely, 406 proteins were significantly downregulated in ACA samples, and the top 16 downregulated proteins are listed in Table 2.

**Fig. 3:**
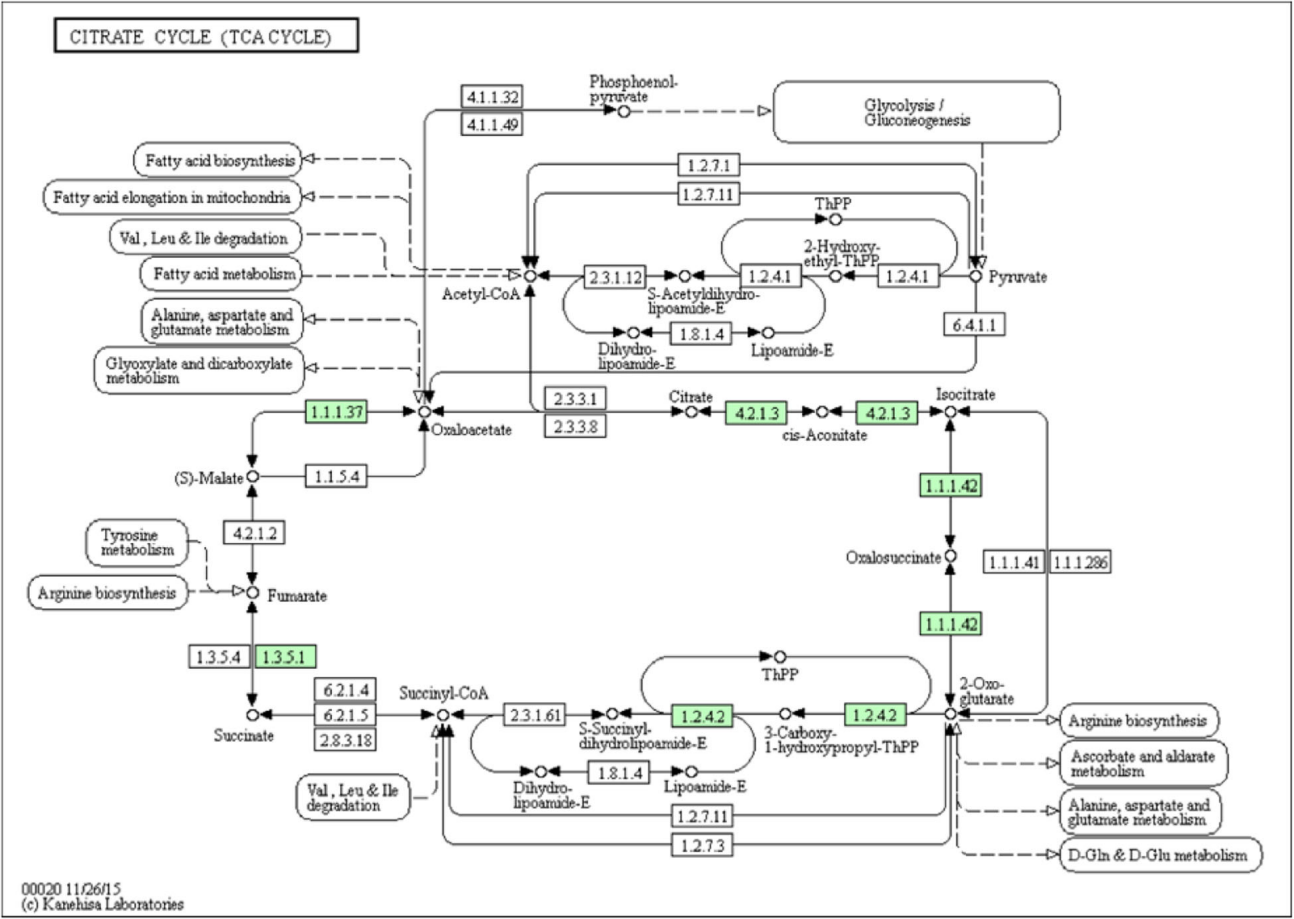
KEGG pathway functional analysis (The numbers represent the ID of proteins in the KEGG pathway, and the green numbers indicate the ID of differentially expressed proteins)

**Table 1:** Top 16 increased expressed proteins in adrenal adenoma compared with normal tissue

Accession	Gene Name	Description	A/B	*P* value
P04259	KRT6B	Keratin, type II cytoskeletal 6B	3.39341	3.45E-24
P08263	GSTA1	Glutathione S-transferase A1	3.29119	4.50E-23
Q499G5	E2F3	E2F3 protein	2.93248	4.08E-19
Q05315	CLC	Galectin-10	2.85897	2.68E-18
P61927	RPL37	60S ribosomal protein L37	2.85584	2.90E-18
P08779	KRT16	Keratin, type I cytoskeletal 16	2.81279	8.77E-18
P04196	HRG	Histidine-rich glycoprotein	2.74085	5.56E-17
A0A0E3DDZ3	HLA-DPB1	MHC class II antigen	2.6654	3.87E-16
Q8NFP4	MDGA1	MAM domain-containing glycosylphosphatidylinositol anchor protein 1	2.34899	1.30E-12
P02741	CRP	C-reactive protein	2.25659	1.37E-11
Q16772	GSTA3	Glutathione S-transferase A3	2.20795	4.70E-11
B2R920		cDNA, FLJ94170	2.11487	4.88E-10
F8TVR8	HLA-DRB1	MHC class II antigen	2.11385	5.01E-10
A7DWG6	HLA-DRB1	MHC class II antigen	2.05556	2.14E-09
Q3T906	GNPTAB	N-acetylglucosamine-1-phosphotransferase subunits alpha/beta	2.03206	3.83E-09
A0A024RB62	METTL1	tRNA (guanine-N(7)-)-methyltransferase	2.02347	4.73E-09

**Fig. 4:**
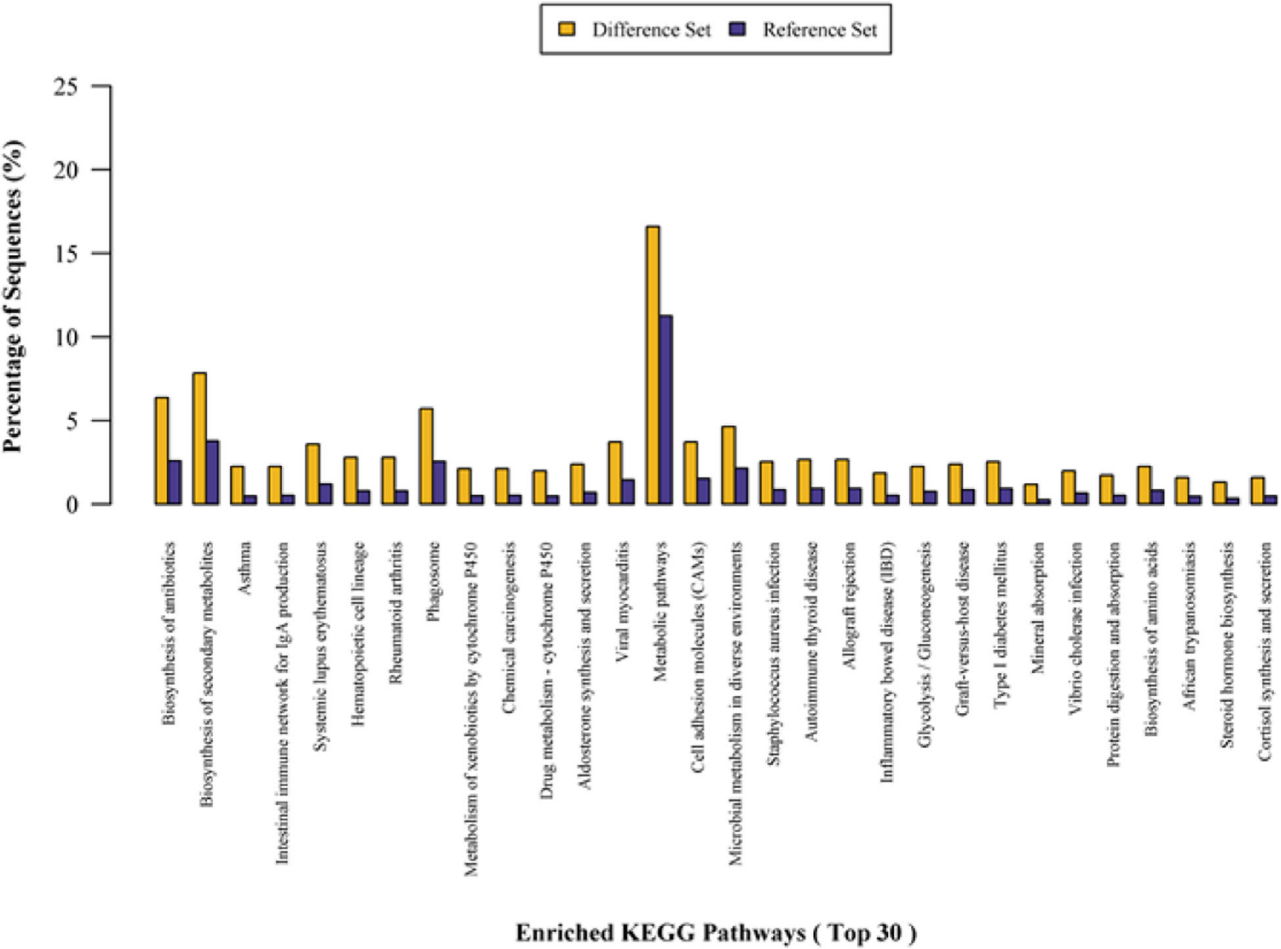
KEGG pathway enrichment analysis (Difference set: target protein set; Reference set: background protein set)

**Table 2:** Top 16 decreased expressed proteins in adrenal adenoma compared with normal tissue

Accession	Gene Name	Description	A/B	*P* value
Q9Y639	NPTN	Neuroplastin	0.789565	0.0499304
I7GW38	ND3	NADH-ubiquinone oxidoreductase chain 3	0.789522	0.0498772
Q9H993	ARMT1	Protein-glutamate O-methyltransferase	0.789151	0.0494224
Q8TDY4	ASAP3	Arf-GAP with SH3 domain, ANK repeat and PH domain-containing protein 3	0.788918	0.049138
Q9NRG7	SDR39U1	Epimerase family protein SDR39U1	0.788899	0.0491147
Q16851	UGP2	UTP--glucose-1-phosphate uridylyltransferase	0.78888	0.0490915
Q6UX07	DHRS13	Dehydrogenase/reductase SDR family member 13	0.788815	0.0490121
Q02978	SLC25A11	Mitochondrial 2-oxoglutarate/ malate carrier protein	0.788382	0.0484886
A0A024QZ64	ALDOC	Fructose-bisphosphate aldolase	0.788057	0.048099
H3BQQ1	CMC2	COX assembly mitochondrial protein 2 homolog	0.787879	0.0478859
P34949	MPI	Mannose-6-phosphate isomerase	0.787826	0.047823
P22748	CA4	Carbonic anhydrase 4	0.787682	0.0476521
Q9BTX3	TMEM208	Transmembrane protein 208	0.787579	0.0475292
A0A0S2Z5N0	BEND5	BEN domain containing 5 isoform 1	0.787255	0.0471465
F2YHL7	APOBEC3F	Apolipoprotein B mRNA editing enzyme cytidine deaminase	0.787215	0.0471
Q49B96	COX19	Cytochrome c oxidase assembly protein COX19	0.786704	0.0465007

### KEGG pathway analysis

To obtain functional pathway information, we further analyzed the DEPs using the KEGG database. KEGG pathway analysis identified the signaling pathways of DEPs (Figs. 3 and 4).

### PPI network of three DEPs

**Fig. 5:**
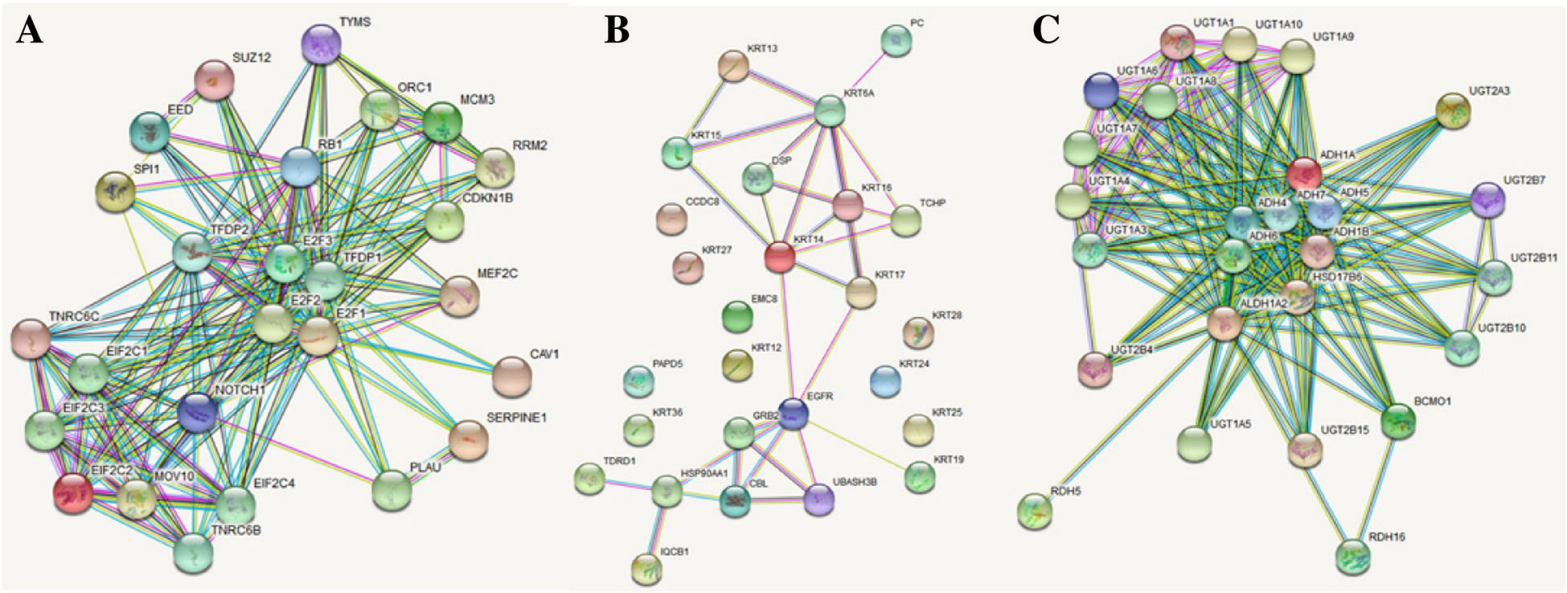
Protein-protein interaction (PPI) network based on the DEPs-. The round nodes indicate individual proteins. Regulations of protein abundance are shown as red (up-regulation) or green (down-regulation) circles. **a** PPI network based on the up-regulated DEP- E2F3. **b** PPI network based on the down-regulated DEP- KRT6A. **c** PPI network based on the up-regulated DEP- ALDH1A2

The interaction network of three DEPs between ACA samples and adjacent normal adrenal gland tissue was predicted using the String database (Fig. 5).

### Verification of three DEPs by Western blot

**Fig. 6:**
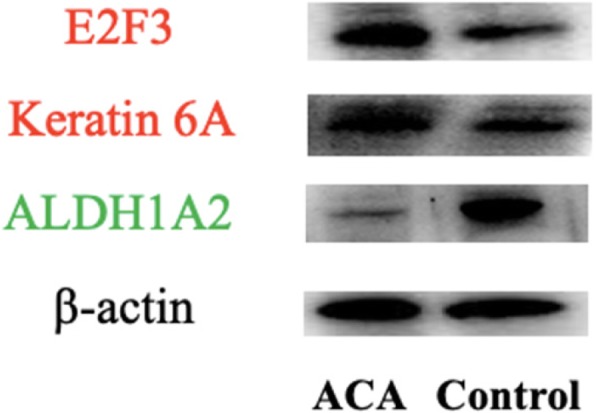
Representative characteristics of adrenocortical adenomas patients for Western blot validation. E2F3 and KRT6A expression increased in ACA samples compared with that in adjacent normal adrenal gland tissue. By contrast, ALDH1A2 expression significantly decreased in ACA samples

We then validated the expression of E2F3, KRT6A, and ALDH1A2 in the abovementioned 24 ACA samples. Western blot analysis revealed that E2F3 and KRT6A expression increased in ACA samples compared with that in adjacent normal adrenal gland tissue (Fig. 6). By contrast, ALDH1A2 expression significantly decreased in ACA samples.

## Discussion

iTRAQ is one of the most advanced technology in modern quantitative proteomics [21, 22]; it combines stable isotope labeling with tandem mass spectrometry [23–25] to compare the relative amount of proteins from normal and diseased samples in a single experiment. Wang WS et al. [26] revealed that myoferlin is a novel prognostic predictor in pancreatic adenocarcinoma through iTRAQ-based quantitative proteomics. In the present study, we used this method to detect protein expression changes in ACAs to identify DEPs that are critical for the molecular mechanism. In particular, we conducted GO and pathway analyses to explore the role of upregulated proteins in ACA progression. Selected DEPs (E2F3, KRT6A, and ALDH1A2) were validated by Western blot. This work will provide a valuable basis for further studies in the field of transformative medicine.

Transcription factor E2F3 is mainly involved in cell proliferation. It participates in transcription repression in quiescent cells by interacting with histone deacetylase and primarily controls genes regulating S phase entry and DNA synthesis. Some studies predicted that E2F3 transcription factor might be a promising biomarker in various cancer and metabolism diseases [27–29]. For instance, to predict overall survival and cause-specific survival in prostate cancer, E2F3 is considered a relatively independent factor [30]. Furthermore, E2F3a stimulates the proliferation of ovarian cancer cells through EGFR-driven mitogenic cell signals [31]. In lung cancer cells, miR-200b can target E2F3 to lessen cell sensitivity to docetaxel [32]. Martinez et al. [33] revealed that E2F3 is involved in DNA damage-induced apoptosis and can regulate the DNA damage response. Thus, E2F3 is a multifunctional factor that is worth further investigations.

Epidermis-specific type I keratin is generally involved in the activation of follicular keratinocytes after wounding, but it does not play a major role in keratinocyte proliferation or migration. Keratin 6A also participates in the regulation of epithelial migration by inhibiting the activity of SRC during wound repair [34]. Chan JKL et al. [35] verified that manipulating K6a phosphorylation or UPS activity may provide opportunities to harness the innate immunity of epithelia against infection. ALDH1A2 as substrates can recognize free retinal and cellular retinol-binding protein-bound retinal. It mainly metabolizes octanal and decanal but does not metabolize citral, benzaldehyde, acetaldehyde, and propanal efficiently [36, 37]. Shou S et al. [38] revealed that defects in IPCD and digit separation in Hoxa13 mutant mice may be partly caused by reduced levels of RA signaling stemming from a loss in the direct regulation of Aldh1a2.

Besides the proteins we mentioned above, some other studies [39, 40] have revealed that immunohistochemistry detecting CYP11B1 and B2 expression was very promising for patients with primary aldosteronism in establishing a final histopathological diagnosis. We also found CYP11B1 in our differentially expressed protein list, but not in the top 16. This might be due to the individual differences. But we can still pay more attention to this procedure, which could be part of the histopathological routine in all operated primary aldosteronism.

In addition, the discrimination of distinct prognosis between ACA and adrenocortical carcinoma (ACC) deserves our close attention. ACA is a curable neuroendocrine tumor that is usually treated via surgery, whereas ACC is a malignant tumor with a low five-year mortality rate and very poor prognosis [41, 42]. Therefore, future proteomic studies may focus on meaningful markers that allow the differentiation between ACA and ACC [6]. An improved understanding of the pathophysiology in these tumors may be obtained by reading lists of hundreds of differentially expressed genes and cellular pathways. There are already some relevant proteomic studies about adrenal cortical tumors [43, 44]. But we still need to go further.

Thus, the PPI network predicted by bioinformatic analysis can provide some useful indications for follow-up scientific research and meaningful clues to explore and detect the binding residues under specific chemical and physical statuses [45–48]. Further improvement is necessary to achieve substantial interactions [49–51]. Developing powerful methods (such as deep neural networks) and obtaining a systematic understanding of the basic mechanisms of PPI require additional time. We hope that the current work will motivate PPI forecasters to conduct further research.

## Conclusions

The iTRAQ technique is a powerful tool for the identification of protein isoforms and comparative proteome studies. In this study, we identified 753 DEPs in ACA tissue compared with the control. Further studies are necessary to understand the functions of the identified proteins (E2F3, KRT6A, and ALDH1A2) in ACAs. A better understanding of the mechanisms underlying the upregulation of these proteins may be important for therapeutic purposes in PA due to ACAs.

## Methods

### Clinical specimens of adrenal adenoma tissue collection

The experimental group randomly collected four clinical specimens of human ACAs from June 2015 to December 2018 in the Second Hospital of Jilin University. The age and gender of all included patients were randomly selected. No adjuvant therapy, such as radiotherapy or chemotherapy, was performed before surgery. ACA tissue was confirmed by pathology after operation. Each patient’s tissue was obtained within 30 min after surgical resection and divided into two parts. One tissue was immersed in 4% formalin solution, and the other tissue was stored in sterile nitrogen tubes in liquid nitrogen. The control group was selected from normal adrenal cortex tissues adjacent to the tumorwhich appears normal under the microscope and was confirmed by pathologists in our hospital (Additional file 1: Figure S1). This experimental study was approved by the Ethics Committee of the Second Hospital of Jilin University.

### iTRAQ

The detailed procedure has been described previously [52]. In brief, the protein samples were precipitated with acetone–TCA and digested by trypsin to generate proteolytic peptides, which were labeled with iTRAQ reagents. The combined peptide mixtures were analyzed by LC-MS/MS for both identification and quantification. Functional enrichment analysis was performed using GO (http://www.geneontology.org/) for biological process, cellular component, and molecular function. Pathway enrichment analysis of protein clusters was performed by KEGG mapping (http://www.genome.jp/kegg/).

### PPI network construction

STRING v10.1 (http://string-db.org/) was applied to analyze the PPI of DEPs identified in the current study and to construct PPI networks. The protein interaction information was extracted from the orthologous proteins of clinical human ACA tissues. The active prediction methods, such as database, experiment, and text mining, were enabled [53].

### Western blot

Proteins extracted from patient samples were separated by 10% SDS–PAGE and then transferred to PVDF membranes (Millipore, Bedford, MA, USA). Membranes were blocked for 1 h in Tris-buffered saline containing Tween (TBST; 20 mM Tris–HCl [pH 7.6], 137 mM NaCl, 0.1% Tween-20) and 5% BSA. After incubation with primary antibodies at 4 °C overnight, the membranes were then washed three times with TBST and incubated with horseradish peroxidase (HRP)-conjugated secondary antibodies (anti-rabbit or anti-mouse IgG: 1:4000, Sigma, USA) for 2 h at room temperature. Bound antibodies were detected by HRP-conjugated rabbit anti-mouse antibody. Band density was quantified by ImageJ and normalized to GAPDH.

### Statistical analysis

Data are given as the mean ± SEM. GraphPad Prism Software (San Diego, CA, USA) was used for statistical analysis. The significance of differences between groups was determined by a non-paired Student’s t-test.

## Additional file

Additional file 1: **Figure S1.** The representative image of medullar-free normal cortex. (DOC 7087 kb)

## Data Availability

All the supporting data are included as additional files.

## Abbreviations

ACA: Adrenocortical adenoma
DEPs: Differentially expressed proteins
iTRAQ: Isobaric tags for relative and absolute quantitation
PA: Primary aldosteronism
PPI: Protein–protein interaction
