# Supplementary figures and images for: ITRAQ-based proteomic analysis reveals possible target-related proteins in human adrenocortical adenomas
Source: BMC Genomics. 2019 Aug 16;20:655. doi: 10.1186/s12864-019-6030-5 (PMC6697928; doi:10.1186/s12864-019-6030-5)

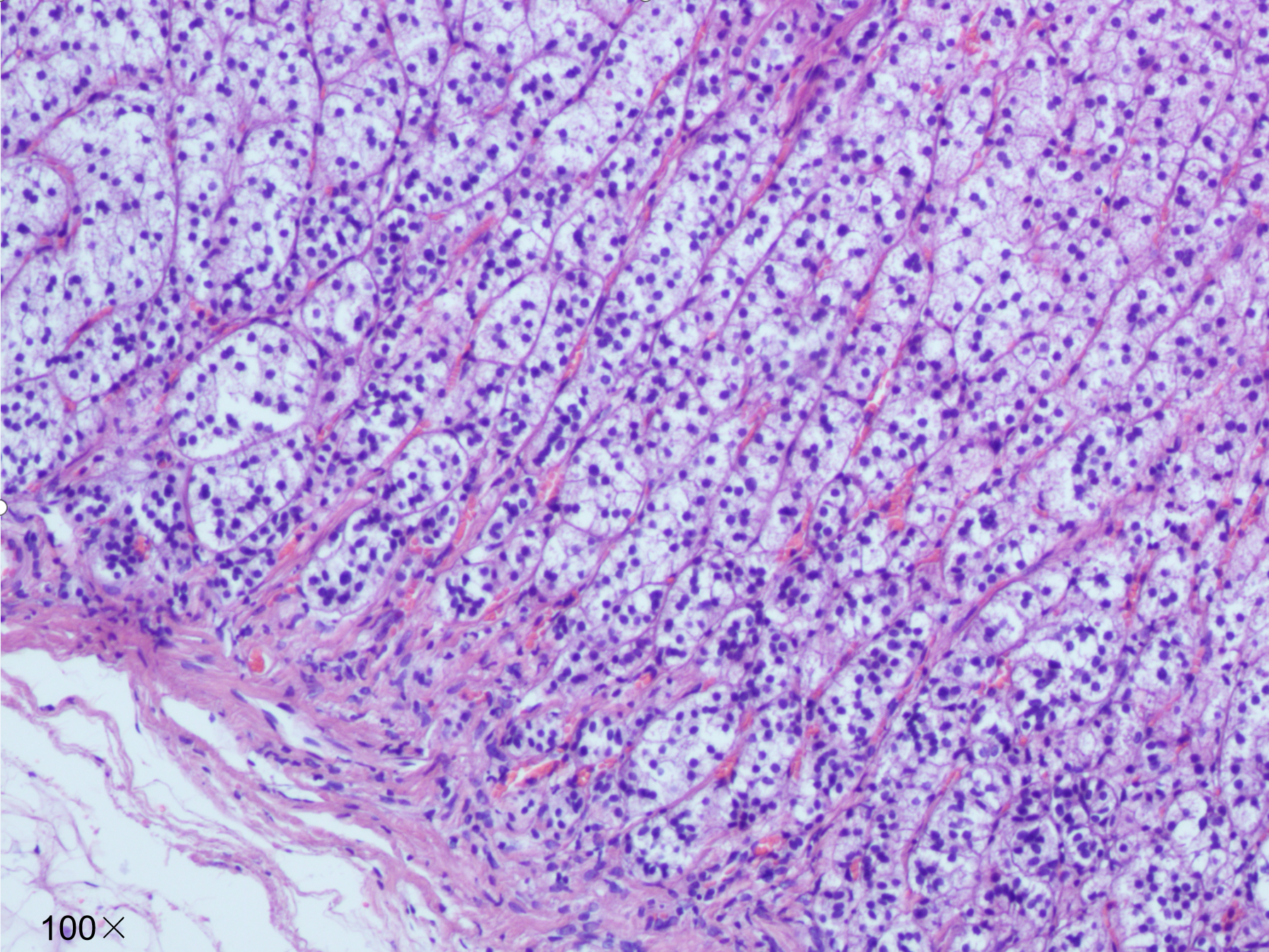
Additional file 1

Figure S1. The representative image of medullar-free normal cortex.

Supplement: Supplementary file 1 — Figure S1. The representative image of medullar-free normal cortex. (DOC 7087 kb) [file 12864_2019_6030_MOESM1_ESM.doc]
